# Mode of production and social formation: a proposal for a new analytical framework based on the concept of socio-economic power

**DOI:** 10.3389/fsoc.2026.1865869

**Published:** 2026-06-25

**Authors:** Stefan Vladimir Mandić

**Affiliations:** Faculty of Philosophy, University of Belgrade, Belgrade, Serbia

**Keywords:** historical sociology, Marxism, mode of production, pre-capitalist societies, social formation, socio-economic power

## Abstract

The article analyzes the relationship between the mode of production and social formation, one of the central, yet most debatable, issues in Marxist theory. The objective is to reconstruct three dominant Marxist perspectives on this subject (reductionist, structuralist, and world-systems) and to propose a new analytical framework. Previous approaches laid important groundwork but did not achieve operational differentiation between the mode of production and social formation as they were still shaped by reductionism, had not yet focused on the issue, or lacked clearly elaborated analytical criteria. In response to these limitations, the article introduces the concept of socio-economic power, defined as the potential of a specific formation’s ruling class to institutionalize and successfully expand its economic, political, and ideological reproduction. Based on five dimensions of socio-economic power (ownership and control over the means of production, surplus appropriation, coercive power, political-legal autonomy, and ideological productivity), a gradational distinction is established between the mode of production, and minor and dominant formations. Furthermore, the article offers a revised classification of pre-modern formations (slavery, patrimonialism, feudalism, and large-scale commodity production) and modes of production (small-scale subsistence and small-scale commodity production). The primary scientific contribution of the article lies in the creation of a flexible conceptual apparatus that enables the analysis of the historical complexity of societies, particularly pre-modern ones, which rarely rested on a single economic system. Rather than offering a closed theory, the article provides an outline of an analytical framework intended for further theoretical and empirical elaboration.

## 1 Introduction

Various schools of Marxism have differently interpreted the concepts of the mode of production and social formation, as well as the interaction between them. The root of this theoretical diversity lies in Marx’s own work. The first, reductionist view was prevalent among Marxists from the time of Marx’s death until the Second World War. The second, structuralist view became dominant from the 1960s onwards. World-systems theory, as a specific strand of neo-Marxist thought, developed a separate theory of the role of modes of labor in the functioning of world systems during the 1970s. The primary objective is to reconstruct the fundamental theoretical positions of these three viewpoints and attempt to propose a new understanding of the mode of production and social formation, along with a related classification of pre-capitalist formations. The focus will not be on a complete reconstruction of these three approaches but rather on the analysis of the segments essential for constructing a new analytical framework. Detailed depictions of the authors belonging to one of the three traditions have an analytical function: to identify the implicit and explicit assumptions regarding the relationship between the mode of production and social formation. The article does not present a fully developed theory but rather outlines an analytical framework for identifying the key dimensions of the relationship between the mode of production and social formations, intended for further theoretical and empirical development. Regardless of the fact that the paper is analytical in character, empirical examples will sometimes be used as heuristic illustrations of the potential of the theoretical framework.

The scientific significance of the article is in the attempt to establish a more adequate classification framework for the historical analysis of the pre-modern societies, thereby contributing to historical sociology. This attempt gains particular significance if one takes into account that pre-modern societies rarely relied on a single economic system and instead represented a mixture of different systems. Classical approaches were shaped by reductionism, had not yet focused on the issue, or lacked clearly elaborated analytical criteria. Therefore, the article introduces the concept of socio-economic power, which enables the differentiation between the mode of production, minor formations, and dominant formations, as well as a more precise understanding of their interaction. The analytical framework is heterodox relative to classic Marxist base-superstructure model. It was created in order to provide an operational tool that could be beneficial when making a distinction between different levels of socio-economic power of various economic systems. Recent works explore various aspects of social formations and modes of production, from digital capitalism, critical analysis of the development of the term mode of production and social formation, to the role of race and patriarchy in reproduction of (early) capitalism, etc., but none provides a clear distinction between mode of production and social formation (cf. Evangelista, 2025; Banaji, 2010; Burns, 2021, 2024; Olson, 2009; Pal, 2020; Pfeiffer, 2022; Souza, 2024; Yan, 2024). It is precisely in this conceptual gap that the relevance of this article lies.

## 2 Reductionist perspective

Before the analysis of the reductionist view, it is necessary to determine what, according to Karl Marx’s most influential view, represents the material base and superstructure. In the *Preface* to *A Contribution to the Critique of Political Economy* Marx (1904, pp. 11–14) is of the opinion that the material base represents the economic foundation of society, which includes productive forces and relations of production. The integral parts of the productive forces are human labor, resources, and means of production (technology) (Cohen, 1978, p. 55). To each stage of development of the productive forces correspond certain relations of production, which are a fundamental class relationship between the main class of exploiters and the main class of the exploited (slave/slave owner, feudal lord/serf, capitalist/wage worker, and centralized state/village commune). The material base of society influences the basic characteristics of the superstructure (law, politics, ideology, and religion) and the general character of society. With leaps in the development of productive forces, old relations of production become obsolete, and the main class of the exploited, through social revolution, creates new relations of production that correspond to the new productive forces, as a result of which a transition from one formation to another is realized (ancient mode of production/slavery>feudalism>capitalism).

For a long time, the most dominant Marxist way of looking at the relationship between the mode of production and social formation was based on the *Preface*. According to this understanding, the mode of production is composed of productive (class) relations and productive forces and, therefore, is implicitly or explicitly identified with the material base. For this viewpoint, a particularly important segment of the *Preface* is the one that emphasizes that different modes of production (Asiatic, ancient, feudal, or bourgeois) designate epochs of social formations (Marx, 1904, p. 14). While the mode of production refers only to the material base, the social formation encompasses the material base/mode of production and the superstructure. Still, since the mode of production is the most important cause of the character of the social formation, these two terms are often used as synonyms. The reductionism of this standpoint is reflected in the frequent position that one mode of production corresponds to one formation and in the opinion that mode of production is equal to the material base.

The question arises whether Marx advocated such a standpoint. The answer is both yes and no, because the real question is not whether Marx advocated a reductionist standpoint, but when. Among numerous authors, there is a debate whether Marx’s thought is coherent or whether irreconcilable gaps exist between its different parts (Althusser, 1969; Marcuse, 1941; Mandel, 1971). During his intellectual work, Marx approached society at different levels of abstraction, which conditioned the appearance of multiple “Marxs” who influenced the development of different schools of Marxism. As an anthropological philosopher, Marx believed that humans as beings of praxis are capable of changing the world (from this standpoint, humanist and libertarian Marxism emerged); as a post-Hegelian, he saw society as a totality in which the influence of different parts cannot be clearly demarcated (this position was the inspiration for Lukacs, Gramsci, the Frankfurt School, and indirectly for structuralist Marxism); as a “scientific” socialist, he searched for general laws of historical development (inspiration for the reductionist Marxism of the Second International (1889–1916), Marxism-Leninism, and Stalinism); as a historian, he studied concrete social formations that were always made up of several intertwined modes of production (like Marxist historians Edward Palmer Thompson and Perry Anderson and structuralist Marxism).

The matter becomes even more complex if one takes into account that Marx often used different levels of analysis and reached different conclusions in the same work. Because of this, every later Marxist could interpret Marx in accordance with their own preferences and interests and the context in which they worked. This is also indicated by the development of the Marxist movement after his death. Most of the continuators of his political line opted for Marx the “scientific” socialist for several reasons. First, to prove that socialist transformation is not a matter of political debate but of historical inevitability. Also, early Marxists wanted to respond to criticisms that socialism is a utopian ideology, making a distinction between Marxism and utopian socialism and insisting on the empirical foundation of Marxism. In accordance with this, Friedrich Engels, in the speech at Marx’s funeral, points out “that just as Darwin discovered the law of development of organic nature, Marx discovered the law of development of human history” (Engels, 1978, p. 681). In *Anti-Dühring*, Engels is of the position that the most important factor of change is the productive forces, while other parts of the social formation only react to changes in technological development. In the same work, Engels equates the mode of production with the relations of production and implicitly with the material base, to which (through the primacy of the productive forces) he gives primacy (Engels, 2021, p. 296). Toward the end of his life, Engels partially departs from the deterministic position, giving limited influence to the superstructure on the character of society (Korolija, 2024, pp. 26–29; Olson, 2009, pp. 184–185).

Engels’s interpretation of Marx influenced the majority of members of the Second International to, to an extent, adopt a reductionist understanding of change. According to this understanding, although the superstructure cannot be completely reduced to a mere reflection of the base, the material base, i.e., the economy, decisively influences the character of society. Karl Kautsky opposes technological reductionism but accepts the position of the economy as the basic cause of the laws of the historical movement of humanity. Therefore, he concludes that the revolution and the transition to socialism are an economic-historical, not a political, inevitability. As the main criticism of the Bolshevik Revolution, Kautsky points out that it happened in Russia, which at that time did not yet have developed capitalism, that is, that the revolution occurred without the basic material circumstances that allow for a true transition from capitalism to socialism (Kautsky, 1918, 1964; Korolija, 2024, pp. 30–31; Olson, 2009, pp. 186–187). Soviet theorists went the furthest in economic and technological determinism after the collapse of the Second International. For Nikolai Bukharin, in society as in nature, there is a law of correspondence according to which one system cannot be explained if it is not understood which parts condition the existence of other parts and the general character of the system. To understand the social system, it is necessary to explain how the development of the means of production affects the character of the economy, politics, ideology, and the overall character of the system (Bukharin, 1925, pp. 25–31; Olson, 2009, pp. 187–188). Joseph Stalin in the work *Dialectical and Historical Materialism* first explicitly equates the material base with the mode of production, claiming that the integral parts of the mode of production are the productive forces and relations of production (Olson, 2009, p. 189; Stalin, 1940, p. 29).

After the Second World War, the reductionist view became less popular among Western Marxists but was still present. Maurice Dobb (1946, p. 7–13) in the *Studies in the Development of Capitalism* holds the position that every historical society is based on one dominant mode of production identified as the economic system (Olson, 2009, p. 191). The mode of production determines the basic characteristics of the entire social system, which is why every epoch of development can be identified with one mode of production. The most developed reductionist view on the relationship between the mode of production and social formation was created by Gerald Allan Cohen. Cohen’s standpoint on important elements abandons the reductionist position, but, due to the insistence on the primacy of productive forces and the reductionist definition of mode of production, it can still, in a wider sense, be classified as reductionist. In Cohen’s view, social formation is understood primarily as a constellation of one dominant and several minor relations of production, and there is no social formation based on only one relation of production. However, the character of the social formation is largely determined by the dominant relations of production (Cohen, 1978, p. 78). Relations of production can, depending on the level of abstraction, be identified with the mode of production because, according to Cohen, Marx defines the mode of production differently at three different levels of analysis:

- Material mode of production—technical characteristics of production; a concept close to productive forces.
- Social mode of production—social aspect: whether production is directed toward profit or needs, based on economic or non-economic coercion, exploits surplus labor or value; a concept close to relations of production.
- Mixed mode of production—the widest level: includes both technical and social configuration; a concept close to the material base (Cohen, 1978, pp. 79–85).

The problem of the reductionist view is the complete or significant abolition of autonomy of the politics and ideology. If the logic of this view was followed, one might expect capitalism to have first developed in pre-modern China given its level of economic and technological advancement (Deng, 2002, p. 2). That not happening would indicate that economic factors are not only ones which are important as political and ideological structures matter as well. The very fact that capitalism started to emerge in medieval Europe even though it was technologically and economically less developed indicate that the understanding of social change in this view is problematic because it neglects the influences of other factors (decentralization of feudalism/centralization of Chinese premodern system, the effect of the political system and ideology on social structures and social change, etc.) (Mandić, 2022, pp. 171–174). The basic weakness of the reductionist approach is therefore the reduction of all characteristics of the social formation to the characteristics of the material base, which is identified with the mode of production.

## 3 Structuralist perspective

In Marx, one can find passages in which the relationship between the mode of production and social formation can be interpreted in a radically different way than they were in reductionist view. The basic passage that became the inspiration for a new reading of Marx by structural Marxists (Louis Althusser, Etienne Balibar, Nicos Poulantzas) is found in the *Grundrisse*: “In all social formations [Gesellschaftsformen] there is one specific mode of production [eine bestimmte Produktion] which predominates over the rest, whose relations thus assign rank and influence to the others” (Burns, 2024, pp. 1–2; Marx, 1973, p. 107). Structural Marxists, in accordance with the cited passage, consider that within one social formation, there are multiple modes of production. A social formation represents a hierarchical articulation of the relationship of a dominant mode of production with one or more minor modes of production. Although the dominant mode of production most significantly determines the character of a social formation, its complex nature cannot be adequately understood if one does not take into account how it is also influenced by minor modes of production (Althusser and Balibar, 1970, pp. 207–208; Poulantzas, 1973, p. 15; Resch, 1992, p. 40).

Besides understanding the formation as a hierarchical articulation of different modes of production, structural Marxists define reproduction in a broader way. In addition to economic reproduction, which is the most important for structural Marxists, legal-political and ideological reproduction are also necessary for the survival of a social formation. The character of the social formation does not depend only on the influence of the economic instance, but it is overdetermined by the joint influence of the economic, political, and ideological instances. Since the reproduction of the social formation is always articulated through the dominant mode of production, structuralists include the economic, the political, and the cultural instance in the wider definition of the mode of production (Althusser, 2014, pp. 21–22; Poulantzas, 1973, pp. 13–17; Resch, 1992, pp. 39; 320–321).^1^ Balibar also gives a narrower definition of the mode of production, according to which it includes three elements (worker, means of production, and non-producer/exploiter) and two relations:

1. the relation of real appropriation, which determines the immediate relationship of the producer toward nature;
2. the relation of property, which indicates in what way the upper class controls the labor process and how it appropriates the surplus (Althusser and Balibar, 1970, pp. 212–213).

When they speak about the economic mode of production, Althusser and Balibar (1970) emphasize that production (class) relations have greater primacy over the productive forces (Milios, 2018, pp. 67–68). When they speak about the general mode of production, they then include political and ideological instances. Balibar calls these instances social structures, defining them as integral parts of the social formation, but also connecting them to the mode of production, highlighting the mode of production as the state of those structures (Althusser and Balibar, 1970, p. 205). It is important to note that in later works Althusser (2014, pp. 21–22) was closer to identifying the mode of production with the economic mode of production, creating a distance toward the broader definition of the mode of production, but leaving the question of the correct definition open. According to structuralists, certain historical formations can be directly more conditioned by cultural and political instances because the character of the economic instance is such that it allows this. Although the economic structure is, in the last instance, the most important, in certain modes of production, it is organized so that its indirect influence is less visible than the influence of politics, ideology, or religion (Harnecker, 1978, p. 24). The main shortcoming of the structuralist position is reflected in the fact that, by expanding the concept of the mode of production, they blurred the difference between mode of production and social formation. Also, structuralists do not give a precise operationalization of how the prevalence of the dominant mode of production within the social formation is measured.

## 4 World-systems perspective

The primary unit of analysis of world-systems theory is not the individual society but the world-system. According to Immanuel Wallerstein, there have been two basic versions of world-systems: the world-empire and the world-economy. World-empires, as bureaucratic systems with a single political structure, integrated different cultures, classes, and societies with different internal economic organizations and financed the state apparatus by taxing different forms of labor and monopolizing trade (Wallerstein, 1974, pp. 15–16, 2004a). World-empires were not interested in the way specific societies and classes within them were organized as long as they regularly paid the rent or tax. According to Wallerstein, the area where the world-empire did not dominate was feudal medieval Europe. European feudalism inherited certain institutions of the Roman Empire but did not have a unified bureaucratic apparatus. Wallerstein (1974, p. 36) defines feudalism as a set of self-sufficient economic nodules based on the direct appropriation of the surplus of agricultural products by the noble class.

A world-economy can be defined as a large geographic space which encompasses multiple cultures, a complex division of labor and significant exchange of goods, but unified economic activity is not accompanied by a single political center, but rather fragmentation into multiple political entities (Wallerstein, 2004b, pp. 23–24). Most pre-modern world-economies did not manage to sustain themselves but were transformed into world-empires over time. Only the European world-economy during the long 15th century (1450–1,550) managed to evolve into a world capitalist system (world capitalist economy). Capitalist world system emerged when the European world-economy successfully integrated different world regions into itself and organized them hierarchically through the existence of the core, periphery, and semi-periphery (Wallerstein, 1974, pp. 67–68). The primary goal of the world capitalist system is the continuous accumulation of capital (Wallerstein, 2004b, p. 24). Relations in the world capitalist system are based on the exploitation of the periphery by the core, while the semi-periphery is both an exploiter of the periphery and exploited by the core. The core bases its exploitation on technological advancement and strong state structures, which allow it to export expensive and technologically advanced products (quasi-monopoly products) and to import cheap raw materials and manufactures (peripheral products) from the periphery (Wallerstein, 2004b, pp. 28–30). Therefore, the dominance of the core is based on the structural organization of the world capitalist system which allows for continuous unequal exchange. Since its emergence, the world capitalist system has integrated different forms of labor into itself. In the early world capitalist system, the wage form of labor dominated in the core, while slave ownership and feudalism were dominant in the periphery (Wallerstein, 1974, pp. 87–100).

Wallerstein avoided the terms mode of production or social formations because they indicate independent and separate social systems. By using the term form of labor (or mode of labor), Wallerstein wanted to signify that within the early world capitalist system, both wage, semi-wage, and non-wage labor were exploited, i.e., that it had the ability to territorially integrate different forms of labor. The main problem of the Wallerstein’s approach is that in the striving for the analysis of global macro-structures, he avoided the level of the specific mode of production or specific social formation, and with the use of the term form of labor blurred the line between social formation and mode of production. Because of this, the question of whether the world capitalist system, capitalist formation, and capitalist mode of production refer to the same or different phenomena remained unanswered.

## 5 Different perspectives on social transformation

Depending on the way they viewed the mode of production and social formation, authors also had different visions of social transformation, namely, the transition from one social formation to another. Marx himself contributed to these different views as he left traces in his work that can point to different conclusions (Mandić, 2024, pp. 43–50). When speaking about the transition from feudalism to capitalism, Marx explained this process in three different ways:

1. In *The German Ideology*, capitalism originates from trade. Merchants invested money accumulated from exchange into manufacturing based on wage labor (Marx and Engels, 2022, pp. 82–83).
2. In the first volume of *Capital*, capitalism emerged in the English countryside through the process of enclosure. The abolition of communal ownership created capitalist agriculture, feudal-capitalists, and a reserve army of labor for industry (Marx, 2010a, pp. 704–738).
3. In Chapter 20 of the third volume of *Capital*, merchants are important because they provided accumulated money and wholesale trade. However, the merchant path is not revolutionary. When merchant capital penetrates pre-capitalist production, it remains primitive. True development emerges only when the producer begins to behave capitalistically (Marx, 2010b, pp. 321–336).

It can be interpreted that Marx believed capitalism, understood in a reductionist sense as a minor mode of production (material base), and appeared as early as medieval Europe, but that it appeared as a pure form of social formation—also understood in a reductionist sense (material base + superstructure)—only with the “capitalist producer” who gradually developed. In his explanation of the transition from feudalism to capitalism, Marx does not strictly adhere to the scheme presented in the *Preface* as it is difficult to apply to concrete historical situations (and in broader Marxism, empirically based theories of transition founded on a reductionist standpoint are rare).^2^ In later interpretations of Marx, either class struggle in the English countryside or the development of commercial cities was taken as the most important cause of the transition to capitalism. The explanation that class conflict is most essential for capitalism was developed by proponents of political Marxism, such as Brenner (2002), Dobb (1946), and Wood (2002). According to political Marxists, the specificities of class conflicts between the English nobility and peasantry led to the establishment of capitalism in England during the 14th and 15th centuries. Another group of authors, such as Sweezy (1976) and, to some extent, Anderson (1974a,b), pays greater attention to the corrosive influence that trade and early (proto)capitalism had on the dismantling of feudalism. A third group of authors, such as Althusser and Balibar (1970) and Milios (2018), advocates an aleatory approach according to which capitalism emerges as a successful incident, when accidental historical circumstances lead to the meeting of the two fundamental classes that constitute it, capital and wage labor, which had developed independently prior to their convergence (this position is in accordance with their opinion that relations od production have primacy over productive forces). Wallerstein (1974) explains the emergence of capitalism through the crisis of European feudalism and the expansion of trade chains, but he does not explain why this process began specifically in Europe, nor how pre-modern trade grew into the specific relationship between capital and wage labor.

## 6 Proposal for a new differentiation between mode of production and social formation

The reductionist approach “solves” the problem of dominance by abolishing the possibility of the coexistence of multiple modes of production. The structuralist approach allows for coexistence but leads to a blurring of the difference between the mode of production and social formation, while the world-systems approach provides an excellent analysis at the macro-level but loses precision at the level of specific societies. The analytical framework of this article will attempt to overcome the shortcomings of these three approaches and thus fulfill three goals: the differentiation of the levels of analysis of the mode of production, social formation, and world-system; the explanation of the coexistence of multiple formations and modes of production; and the explanation of the dominance of a specific formation.

If a certain mode of production exists continuously at the economic level, it inevitably produces political and ideological instances. The interaction of economic, political, and ideological structures leads to the mode of production establishing itself not only as an economic form but also as a way of life and a worldview. The question arises: if the mode of production is a separate entity that continuously reproduces its own economic, political and ideological structures, how does it differ from a social formation? What distinguishes a mode of production from a formation is that a formation always implies that a dominant class has the capacity to extend influence to external territories and broader social structures, i.e., to possess socio-economic power. Socio-economic power can be thus defined as the potential of formation’s ruling class to institutionalize and reproduce its patterns of surplus appropriation and labor organization in a manner that enables stable influence and expansion into external social structures and territories.^3^ The primary source of socio-economic power is the economic instance, but the interaction of the economic, ideological, and political instances is necessary for its continuous reproduction. Socio-economic power does not always mean the control of the entire society, but rather of the structures that are crucial for its general reproduction. To more precisely determine the difference between a mode of production, a minor formation, and a dominant formation, it is necessary to operationalize the dimensions of socio-economic power of the ruling class in a given formation or mode of production (or the primary group, in cases where no ruling class exists, such as with the peasantry in subsistence production). The distinction between a mode of production, a minor formation, and a dominant formation stems from the dimensions of socio-economic power. Each dimension is measured using indicators with the following scores: 1 (partial capacity), 2 (stable capacity), and 3 (systemic capacity). Score 0 is implicit indicator which points to absence of capacity and appears primarily in non-economic dimensions. The dimensions are as follows:

1. Ownership over the means of production (economic instance)—whether the class has ownership over the means of production (1—has no ownership, but has a right of use or is a smallholder; 2—owns substantial assets which are still not a majority of means of production within the society; 3—has ownership over the majority of the means of production within the society);
2. Form of surplus appropriation (economic instance)—whether the class has control over the surplus (1—once obligations to other classes are met, it has control over a portion of the surplus; 2—has majority control over surplus appropriation; 3—the way the class appropriates the surplus is dominant in society);
3. Coercive power (political instance)—whether the class has control over armed forces (1—possession of local forces that are subordinate to the center; 2—possession of independent forces; 3—possession of forces that can defeat all other classes within the society);
4. Political-legal autonomy (political instance)—whether the class has its own courts and ability to (self)govern (1—existence of courts for internal disputes while more serious cases go before a central court; existence of political bodies only for the partial internal self-governing; 2—the class possesses an independent judiciary and ability to govern via political bodies on a limited territory; 3—the form of judiciary and government used by the class is the most dominant in the entire society);
5. Ideological productivity (ideological instance)—whether the class is capable of creating a discourse that can spread even to areas not under its direct control (1—creation of a local discourse dependent on the ruling ideology; 2—creation of a coherent discourse that has its own institutions and the capacity for social expansion; 3—control over a discourse that is socially dominant and determines what is considered desirable behavior in society).

The dimensions are not strictly quantitative but should be understood as ordinal heuristic tool. The scale does not aim to provide precise measurements but to capture the potential of a specific mode of production or formation for expansion. The dimensions necessarily stem from the theoretical model of the three instances, with the goal of showing that the reproduction and expansion of a mode of production or formation do not depend only on the economy but on the mutual influence of the three instances. The minimum condition for the existence of a mode of production is at least initial control ownership of the means of production (1) which is why any social form that does not meet this condition does not qualify as a mode of production. The dimensions of a mode of production gravitate around score 1. A mode of production, therefore, has total dimension scores ranging from 1 to 5. The minimum condition for the existence of a minor formation is 2 for the ownership dimension, 2 for surplus appropriation, and 2 for coercive power. The dimensions of a minor formation gravitate toward the score value of 2, with the possibility that certain dimensions may be 1 or even 0 (in some occasions for ideological productivity). The range of scores values for a minor formation is therefore 6–10. The minimum condition for the existence of a dominant formation is at 3 for the dimension of ownership and control over the means of production, 3 for surplus appropriation, 3 for coercive power, and at least 2 for political-legal power (without stable political and judicial institutions (scores 2 or higher) there cannot be continuous reproduction of dominance). The dimensions of a dominant formation gravitate toward the score of 3, with the possibility that one or more dimensions may have scores of 2 or 1 and rarely 0 (in very rare occasions of crisis or forming of ideological productivity).^4^ The range of scores for a dominant formation is therefore 11–15. The thresholds by which we distinguish between a mode of production, a minor formation, and a dominant formation may seem *a priori*. It is important to note that they arise from the logic and the conceptual coherence of the model, not from empirical reality, as the model has not yet been empirically applied. This classification cannot encompass the entire complexity of empirical historical reality but can provide framework for its better understanding. Precise dimension scores for the mode of production, minor formation, and dominant formation are shown in Table 1.

**Table 1:** Values of the dimensions of socio-economic power for the mode of production, minor formation, and dominant formation.

Dimensions of socio-economic power	Mode of production	Minor formation	Dominant formation
Ownership over the means of production	1	2	3
Form of surplus appropriation	0–1	2	3
Coercive power	0–1	2	3
Political-legal autonomy	0–1	0–2	2–3
Ideological productivity	0–1	0–2	0–3
General level of socio-economic power	1–5	6–10	11–15

The difference between a mode of production, a minor formation, and a dominant formation is gradational, not by their essence. They differ according to the degree of socio-economic power or the fulfillment of the dimensions of socio-economic power. From all the above, the following definitions of basic concepts emerge:

1. Mode of production (mode of life) represents a coherent unity of economic, political, and ideological instances based on a stable organization of labor, and sometimes on a fundamental class relationship, enabling the reproduction, but without the existence of external socio-economic power (only the existence of internal power for inner regulation) and the capacity for expansion (basic reproduction). A mode of production can also be defined as a reproductive unity whose economic, political, and ideological instances have a primarily internal function.
2. Minor formation represents a unity of economic, political, and ideological instances based on a stable organization of labor and a fundamental class relationship with significant socio-economic power and capacity for expansion on external territories and social structures (extended reproduction).
3. Dominant formation represents a unity of economic, political, and ideological instances based on a stable organization of labor and a fundamental class relationship with hegemonic socio-economic power, a pronounced capacity for expansion and a decisive influence on social structures (systemic reproduction).

In this paper, the mode of production is conceptualized as any form of organized production, which does not necessarily presuppose a fundamental class relationship or the power of one class over another. In this way, the concept of mode of production can encompass subsistence production and small-scale commodity production. It should be noted that every social formation is a mode of production but that not every mode of production is a social formation. What distinguishes a mode of production from a formation is that a formation must be based on a fundamental class relationship, whereas a mode of production does not need to be. The definition of the mode of production is close to Marx’s characterization of mode of production as a “definite mode of life” (Marx and Engels, 2022, pp. 7–8) in *The German Ideology*.^5^

A priority of the dimensions of economic instance over the dimensions of political and ideological instances is assumed, and it could be expected for the economic instance to be established first. However, it is important to note that successful reproduction would not be possible without interaction of all three instances. The possibility of borderline cases is not excluded, in which, for example, ideological or coercive power may be high while surplus appropriation or ownership and control over the means of production are non-existent. However, it is expected that empirical research will show such cases to be rare. In structuralist Marxism, subordinate forms of the mode of production often have a positive function in the reproduction of the dominant mode of production and the social formation. Here, the position will be held that a minor formation or mode of production can, in different historical cases, be functional, but also non-functional and dysfunctional (corrosive) for the reproduction of the dominant formation.

The categories of mode of production and social formation are analytical constructs that simultaneously represent theoretical tools and designations for empirically observable, relatively stable patterns of social relations. The mutual interaction of the dominant, minor formation, and mode of production can occur within a single society, a cluster of societies, or a world-system. An example of a single society would be pre-modern Tokugawa Japan (Hane, 1991, pp. 136–139), in which feudalism was the dominant formation which, due to geographical and political limitations (the isolation of Japan as an island; the strength of patrimonial formations in China and Korea), could not expand into new territories. A cluster of societies would represent multiple societies that, due to mutual civilizational connection and efficient diffusion, share a dominant social formation, such as the slave-holding cities of ancient Greece and the feudalism of medieval Europe (Anderson, 1974a, pp. 30–213). World-systems can include large economic entities that encompass significant parts of the planet, such as world-empires and the world capitalist system. A world-empire has the capacity to integrate several different formations and modes of production into itself. The world capitalist system is based on capitalism as the dominant formation and also has a great capacity for the integration of non-capitalist minor formations and modes of production within the periphery and semi-periphery (this was especially the case in the early world capitalist system). The concepts of core, semi-periphery, and periphery are not the same as social formation and mode of production, but they indicate which structural position individual modes of production and social formations occupy within the world capitalist system. A specific society, cluster of societies, or world-system can, therefore, be understood as a sum of one dominant and one or more minor social formations and modes of production. A society, cluster of societies, or world-system is named after the dominant formation (e.g., if the dominant formation is feudal, the society will also be called feudal).

The theoretical assumption regarding the existence of multiple social formations and modes of production within a single society, cluster of societies, or world-system is particularly heuristically valuable for the study of pre-modern societies in which the hierarchical presence of a dominant formation and one or more minor formations was very frequent. In accordance with the theoretical assumption of the coexistence of multiple formations within a single society, a modified classification of pre-modern formations from the authors doctoral dissertation will be presented here (Mandić, 2022, pp. 158–162). It is important to note that this classification is not considered as completely finished and that criticisms from the wider scientific community could improve it. Furthermore, it is important to point out that this classification only contains formations with a defined class division, while it does not include stateless societies and modes of production that have organized forms of labor but do not have a fundamental class relationship. The basic classificatory framework of pre-modern social formations is based on three dichotomies representing the systemic characteristics of a specific formation:

1. Production for needs/production for the market;
2. Exploitation of slave labor/exploitation of dependent peasantry (through tithe)/exploitation of free peasantry (through taxation);
3. Centralization (existence of an organized hierarchical state apparatus)/decentralization (independent regional units perform most economic, legal, and military functions).

Based on these dichotomies, four pre-modern social formations can be determined which managed to establish themselves as dominant at an empirical level and often existed as minor in certain historical situations:

1 Slave-ownership—A social formation in which the exploitation of unfree labor is carried out. It includes two sub-types:

- Decentralized slave-ownership—Ownership of slaves dispersed among citizens of different class affiliations. Although the upper class owns a larger number of slaves, it is common for the middle and lower classes to own slaves as well. The dispersion of slave ownership also leads to dispersed political power and democratic political order. This formation was dominant in ancient Athens (Anderson, 1974a, pp. 30–33; 35–40; Mandić, 2022, p. 158).
- Centralized slave-ownership—In the first type of this formation, corporately organized citizens have collective ownership over slaves through a centralized state—dominant in ancient Sparta. In the second type, due to stratification among free citizens, ownership of slaves is concentrated in the hands of the upper class (patricians), which affects the creation of a strong state apparatus in order to serve its interests—dominant in Rome during the late republic and principate phases (Anderson, 1974a, pp. 33–34; 53–103; Mandić, 2022, pp. 158–159).

2 Patrimonialism—Definition of patrimonialism in this paper is close to Weber’s (1978) understanding of patrimonialism, Marx’s understanding of the Asiatic mode of production, and Amin’s (1974, pp. 98–100) understanding of the tributary mode of production (Anderson, 1974b, pp. 473–483; Marx and Engels, 1972, pp. 33–37; Pešić, 2025, p. 168). Patrimonialism is, as a rule, the dominant formation within world-empires. In this formation, a centralized state as the ruling class, through a permanent bureaucracy and standing army, exploits the surplus product of the free peasantry or dependent peasantry through taxation/tribute. Typical examples of patrimonialism in which the state exploits the labor of free peasants through taxation are ancient Egypt, pre-modern China, and 8th century Byzantium (Antonić, 1995, pp. 262–265; Deng, 2002, pp. 98–100; Weber, 1978, pp. 1044–1,051). The formation in which a centralized state exploits the labor of dependent peasants through tithe is characteristic of Byzantium during the colonate (Antonić, 1995, p. 258). Centralized slave-ownership, due to the common existence of a centralized bureaucracy and professional army, shares many similarities with strong patrimonialism.
3 Feudalism—A decentralized formation in which surplus product of dependent peasants is exploited by a decentralized noble class. Each individual nobleman, through ownership of a feudal estate, achieves economic, political, and military independence, which leads to the parcellization of sovereignty (Anderson, 1974a, p. 150), i.e., to the fact that central power in the state is chronically weak. Due to the economic independence of feudal estates, most political functions in feudalism, unlike in patrimonialism, are not in the hands of the central state but at the local level. The most famous example of developed feudalism is medieval Europe (Anderson, 1974a, pp. 94–179). The formation was also highly developed in pre-modern Japan (Hane, 1991, pp. 136–139).
4 Large-scale commodity production—A pre-modern mode of production that comes closest to capitalism. Its primary goal is to achieve profit using non-capitalist methods. Therefore, profit is achieved by selling goods on the market that were not produced through the exploitation of wage labor but through the exploitation of slave or serf labor or by selling goods whose price is high due to monopoly control, rarity, or long-distance trade. Present as a minor formation in Rome, Ancient Greece, and medieval Europe (Milios, 2018, pp. 104–120).

Factors leading to the emergence and disappearance of formations are numerous (climate change, demographic trends, technological innovations, class conflicts, diffusion of institutions, and geopolitical competition) (Mandić, 2022, pp. 174–176). Their detailed analysis goes beyond the scope of this article, whose primary goal is conceptual-classificatory (synchronic), and only secondarily dynamic-explanatory (diachronic). Future analysis should strengthen the diachronic part of the conceptual framework and, besides the disappearance and emergence of formations, better explain how the mechanism of transition from a mode of production to a minor formation and from a minor to a dominant one works. Besides the four primary social formations, two modes of production should also be mentioned, characterized by the existence of an organized form of labor but also the non-existence of a fundamental class relationship.

1. Small-scale subsistence production—A mode of production in which free or dependent peasants, often after satisfying the needs of higher classes or the state, satisfy their own needs through independent labor. As a rule, it is present alongside feudalism or patrimonialism as the dominant formation. Present as a significant mode of production in the 18th century and the first half of the 19th century in the northeastern parts of the USA (Korolija, 2026).
2. Small-scale commodity production—A mode of production in which free peasantry and artisans continuously produce for the market. Often goes alongside patrimonialism (Deng, 2002, pp. 195–197). The peasantry, when it satisfies its needs and fulfils obligations to the state, sells the surplus product on the market. Also present in medieval Europe as a form of behavior of the dependent peasantry after meeting obligations to the feudal lord and as the production of guilds and crafts.

It should be noted that in certain historical cases, no single formation may be dominant. According to Sweezy (1976), after the decline of feudalism in 15th-century, a transitional period emerged in which neither feudalism nor capitalism was dominant, but rather pre-capitalist commodity production. Contemporary Marxist historian Yazdani (2017, pp. 21–22) similarly argues that “parts of late 16th to late 18th century Mughal India and its successor states were in a transitory phase where different modes of production coexisted with each other” Yazdani (2017, p. 20) without any one mode being dominant. Yazdani (2022, p. 487) also offers the French Caribbean colony of Saint-Domingue in the 18th century as an example of a society lacking a single dominant mode of production, describing it as a transitional “semi-capitalist slave-based mode of production”.^6^^,^^7^ In order to improve this model in future research, it will need to take into account historical periods in which no single economic form is dominant.

Empirical historical societies were mostly comprised of a combination of multiple formations and modes of production. In pre-modern China (e.g., Han dynasty), strong patrimonialism as the dominant formation had a score of 15 (each dimension 3: state ownership of land, taxation as surplus appropriation, standing army, centralized courts, and Confucian ideology). It was accompanied by the small-scale subsistence and commodity production of the peasantry as modes of production (total score 2–4: ownership 1—peasant works own plot; surplus 1—only small portion left after tax; coercion 0—no own forces; courts 0–1—no local jurisdiction or limited jurisdiction; ideology 0–1—no independent discourse). In certain historical periods such as the Waring States, feudalism temporarily became the dominant formation and had a score of 13 (ownership 3—lords hold land; surplus 3—feudal rent; coercion 3—independent armies; political-legal autonomy 2—own courts but nominal central authority; ideology 2—localized legitimacy). Similar minor feudal formation (6–10) appeared during The Six Dynasties Period (220 CE–589 CE) and The Five Dynasties and Ten Kingdoms Period (907 CE–979 CE).

In medieval Europe, feudalism as the dominant formation had a score of 14 (ownership 3—nobility holds most land; surplus 3—feudal rent is dominant; coercion 3—independent vassal armies; political-legal autonomy 2—manorial courts share power with royal courts; ideology 3—Church provides hegemonic discourse). Feudalism was accompanied by the subsistence and commodity production of the peasantry as modes of production (total score 2–3: ownership 1—peasant works own plot or has a right to use a plot for personal needs; surplus 1—only small portion left after rent; coercion 0—no own forces; courts 0–1—no local jurisdiction or limited jurisdiction; ideology 0—no independent discourse) and by large-scale commodity production as a minor formation (total score 9: ownership 2—merchant capital controls workshops and ships; surplus 2—significant control over the profit; coercion 2—urban militias; political-legal autonomy 2—city statutes and courts; ideology 1—early humanism, not yet developed). Large-scale commodity production later mutated into capitalism and gradually weakened feudalism (from 9 toward 11–15). The leap in agricultural productivity during the 11th and 12th centuries contributed to the monetization of the European economy (for the sake of exchanging agricultural surpluses) (Mann, 1986, p. 378), the accumulation of commercial capital, the development of the banking sector, and the expansion of commodity production oriented toward profit. Soon, capitalism has a fundamental class relationship between the bourgeoisie and the proletariat in free cities (Milios, 2018, pp. 170–180; Nicholas, 2013, pp. 106; 131–132) and had multiple negative impacts on feudalism. It drew labor from the feudal hinterland, dismantled the feudal estate and the guild through competitive production and further monetization, made the noble class dependent on luxury consumption, led to significant debt for the most powerful nobles, created the first non-feudal bureaucratic cadres, and significantly contributed to the development of anti-feudal ideological discourses (humanism, Renaissance, scientific method, and Protestantism) (Mandić, 2022, pp. 182–199; Pirenne, 1946, pp. 221–231; Sweezy, 1976). All these processes led to the gradual strengthening of the power of the capitalist class and the centuries-long evolution of capitalism from a minor to a dominant formation.

The example of the relationship between European feudalism and capitalism provides important elements for future diachronic analysis. The relationship between feudalism and capitalism indicates that social transformation is often not caused by a conflict of classes within one mode of production or social formation, but by the corrosive influence of a minor formation and a conflict between the ruling classes of the minor and dominant formations. Certain minor formations can have a particularly dysfunctional (corrosive) influence on a dominant formation, such as the influence of feudalism on patrimonialism and, especially, the influence of capitalism on feudalism. The world capitalist system as an external factor can also influence the collapse of a specific dominant formation (the world capitalist system had a decisive influence on the dismantling of Japanese feudalism and Chinese patrimonialism). The reverse process is also possible, where a certain type of dominant formation limits the development of a minor formation. Patrimonialism, as a centralized formation, by limiting the independence and economic power of merchants, prevented the mutation of large-scale commodity production into capitalism.^8^ Unlike patrimonialism, in feudalism, power vacuums emerged due to the parcellization of sovereignty within which capitalism could develop.

## 7 Conclusion

The primary advantage of the model presented in this paper over the three Marxist viewpoints is multifaceted. Unlike the reductionist position, it insists on the high autonomy of the ideological and political spheres. In contrast to the structuralist position, which did not make a clear distinction between the mode of production and social formation, our work introduces socio-economic power as the key difference. Finally, unlike the world-systems approach which neglected specific societies, model enables the analysis of the complex interaction of all levels of the system: from the mode of production, through the minor formation, to the dominant formation and the world-system. It is worth underlying that approach in the paper is heterodox relative to classic Marxist base-superstructure model and is preliminary heuristic tool open for further critique and refinement. Although this analytical framework brings a new perspective to the analysis of the relationship between modes of production and social formations, it has not yet been fully tested and as such carries a series of unresolved questions. Regardless of potential limitations (heterodox definition of mode of production and social formation, the arbitrariness of the thresholds, the insufficiently clear determination of transitional hybrid phases, etc.), the theoretical framework of the article establishes the foundations for a flexible conceptual apparatus that will enable studying societies in their historical complexity. The basic heuristic values of analytical framework are multiple. It provides analytical distinction between the mode of production and social formation. Also, it is making possible to explain the coexistence of multiple modes of production and social formations. Besides that, it creates criteria for the hierarchization of different modes of production and social formation and existence of a developed typology of pre-capitalist formations and modes of production. Potentially, it also provides basis for developed diachronic analysis and more adequate explanation of social transformation.

## Footnotes

1 “By the term *mode of production,* Althusser and Balibar (1970) mean not only the forces and relations of production but also their social conditions of existence and the reproduction of those social conditions. Thus, the concept of a mode of production refers not simply to the economic instance…but also…non-economic instances are essential to the reproduction of the forces and relations of production. While the term *mode of production* is less comprehensive than the term *social formation*, it is more comprehensive than the economic instance alone” (Resch, 1992, p. 39).

2 Mohajer and Yazdani, (2022, p. 179; 184–185; 197–199; 207) had a more nuanced view on the transition to capitalism. He was aware that empirical capitalism emerged as unevenly developed process, considering it as a “combination of different moments of original accumulation” (Mohajer and Yazdani, 2022, p. 237). Hence, he took into account how various internal and external factors contributed to the causal process of emergence of capitalism: enclosers, state policies, development of credit system, development of technology, trade policies, colonization and slavery, etc.

3 The definition of power in this work differs from earlier definitions of power in historical sociology. Unlike Weber (1978), who defines power as the ability to influence someone regardless of whether consent is given, and Mann (1986) and Elias (2000), who define power primarily through the existence of power networks or chains of interdependence, the concept developed here emphasizes the structural ability of a class to reproduce and expand its form of economic appropriation, its political and ideological institutions.

4 In post-communist Eastern Europe during the early stages of the formation of the new system, capitalism without capitalists was not based on a distinct ideology of a capitalist class, which was non-existent, but rather on the technocrats of the former socialist regime whose rule was not founded on “grand narratives” but on expertise and power (Eyal et al., 1998). Military juntas in Latin America were also not capable of creating an ideological matrix that could penetrate the masses. Instead, they ruled through bureaucracy and military force.

5 “The way in which men produce their means of subsistence depends first of all on the nature of the actual means of subsistence they find in existence and have to reproduce. This mode of production must not be considered simply as being the production of the physical existence of the individuals. Rather it is a definite form of activity of these individuals, a definite form of expressing their life, a definite mode of life on their part” (Marx and Engels, 2022, pp. 7–8).

6 Furthermore, author describes 16th-to-18th-century French economic system as a “transitionary phase where feudal (seigniorial), commercial capitalist and entrepreneurial elements coexisted” (Yazdani, 2022, p. 465).

7 Following the War of Independence Saint-Domingue was reconstituted as Haiti, and the economic system in the former colony became less capitalist and more contradictory, marked by elements of subsistence production, foraging and barter (Yazdani, 2022, pp. 458; 494).

8 Equating all Asian and other non-European societies with Asian mode of production or patrimonialism is Europocentric and is not supported in this article. The non-emergence of industrial capitalism in different Asian societies cannot in every case be explained by patrimonialism alone. In 18th-century in Gujarat, there was a developed merchant class and sophisticated private production of textile, dyes, and ships (Yazdani, 2017, p. 63). However, the development of autochthone capitalism was prevented by a number of unfavorable circumstances (caste, ethnic and religious divisions among merchants, lack of military independence, the absence of secular educational institutions, and economic disincentives to innovate) (Yazdani, 2017, pp. 416; 427–428; 516; 554–555). Unlike the English bourgeoisie, which enjoyed the support of a centralized state, in Gujarat the fragmentation of political authority (born out of struggles between different factions of the landlords, the piracy, and incursions by the Marathas and British), not patrimonialism, made undisturbed capital accumulation difficult (Yazdani, 2017, pp. 181; 196; 562).
